# 4,5,6,7-Tetrahydroindol-4-Ones as a Valuable Starting Point for the Synthesis of Polyheterocyclic Structures

**DOI:** 10.3390/molecules26154596

**Published:** 2021-07-29

**Authors:** Tomas Horsten, Wim Dehaen

**Affiliations:** Molecular Design and Synthesis, Department of Chemistry, KU Leuven, Celestijnenlaan 200F, B-3001 Leuven, Belgium; tomas.horsten@kuleuven.be

**Keywords:** pyrrole, indolone, polyheterocycle, natural product, biological activity, optoelectronics

## Abstract

This review focuses on the synthesis of polyheterocyclic structures with a variety of medicinal and optoelectronic applications, starting from readily available 4,5,6,7-tetrahydroindol-4-one analogs. First, routes toward the 4,5,6,7-tetrahydroindol-4-one starting materials are summarized, followed by synthetic pathways towards polyheterocyclic structures which are categorized based on the size and attachment point of the newly formed (hetero)cyclic ring.

## 1. Introduction

**Figure 1:**
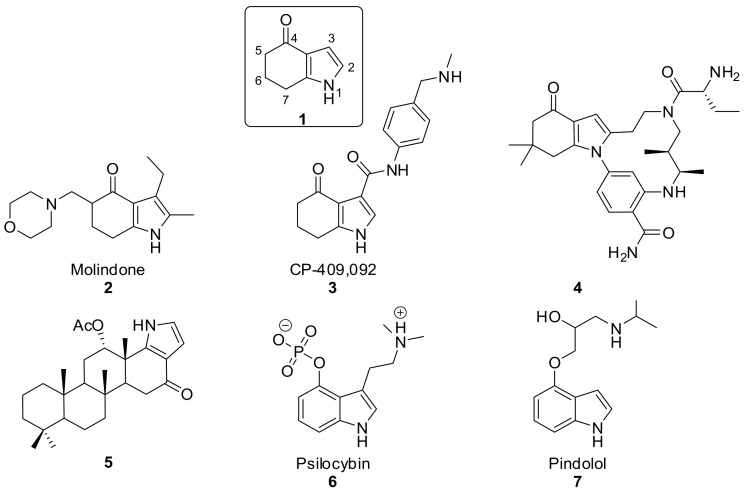
4,5,6,7-tetrahydroindol-4-one **1** motif in drug design and natural products.

Pyrrolocyclohexanones, such as 4,5,6,7-tetrahydroindol-4-one **1** and their derivatives are important structural motifs found in a variety of drugs, for example the FDA approved antipsychotic molindone **2** used to treat schizophrenia [1], the GABA_A_ agonist CP-409,092 **3** for the treatment of anxiety [2], and the potent heat shock protein 90 (Hsp90) inhibitor **4** for cancer treatment [3] (Figure 1). Examples of natural occurrence of **1** derivatives are scarce. However, the pyrrolo fused scalarane sesterterpenoid **5** has been isolated from sponges [4]. Furthermore, dehydrogenation of the 4,5,6,7-tetrahydroindol-4-one structure gives rise to a 4-hydroxy-indole moiety which is found in many bioactive alkaloids such as psilocin and its precursor psilocybin **6** [5] and FDA approved drugs, for example the non-selective beta blocker pindolol **7** [6].

Due to the presence of the pyrrole and ketone functionality, 4,5,6,7-tetrahydroindol-4-one analogs have received considerable attention for the synthesis of complex polyheterocyclic structures with a variety of medicinal and optoelectronic applications. This review will focus on the synthesis of polyheterocyclic structures starting from 4,5,6,7-tetrahydroindol-4-one analogs. First, routes toward the 4,5,6,7-tetrahydroindol-4-one motif are summarized, followed by synthesis of polyheterocyclic structures which are categorized based on the size and attachment point of the newly formed (hetero)cyclic ring.

## 2. Synthesis of 4,5,6,7-Tetrahydroindol-4-One Analogs

**Scheme 1:**
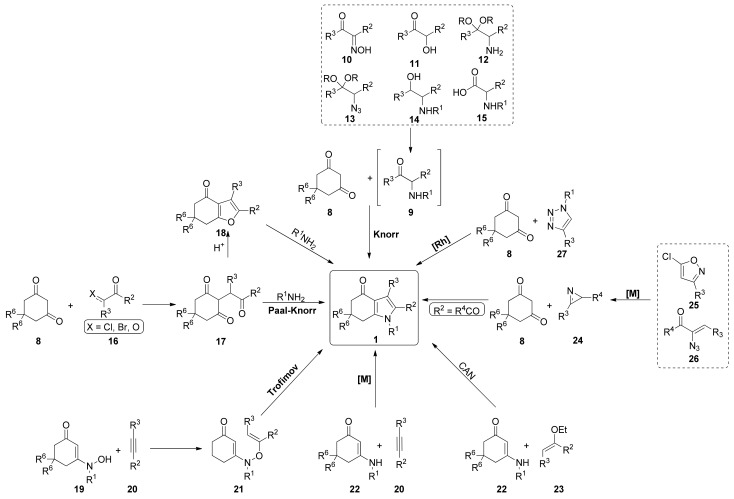
Synthetic routes toward 4,5,6,7-tetrahydroindol-4-ones **1**.

The synthesis of the 4,5,6,7-tetrahydroindol-4-one motif **1** was first reported in 1928 by Nenitzescu and Scortzbanu, starting from condensation of 1,3-cyclohexadiones **8** with *α*-aminocarbonyls **9** in a [2+3] fashion (Scheme 1) [7]. Since many *α*-aminoaldehydes **9** (R^3^ = H) and *α*-aminoketones **9** (R^3^ ≠ H) self-condense easily, precursors have been used to form *α*-aminocarbonyl **9** building blocks in situ. Nenitzescu and Scortzbanu therefore introduced the amine functionality by reduction of oximes **10** with zinc as described in the classical Knorr pyrrole synthesis [8]. Later on, these *α*-aminocarbonyl building blocks **9** have been synthesized from various other precursors. Thus, *α*-hydroxy ketones **11** have been condensed with ammonium acetate [9]. The aldehyde or ketone functionality can be formed from *α*-aminoacetals **12** [10,11] and *α*-azidoacetals **13** (aza-Wittig reaction) [12], oxidation of *α*-aminoalcohols **14** [13,14,15,16], or from dehydration of amino acids **15** [17,18]. Stetter and Siehnhold reported the synthesis of **1** starting from alkylation of 1,3-cyclohexadione **8** with phenacyl bromide **16** (X = Br, R^2^ = Ph, R^3^ = H) followed by a Paal-Knorr pyrrole synthesis of triketone **17** with primary amines [19]. Over the years, many modifications have been applied of this [4 + 1] strategy including different *α*-haloketones **16** and different nitrogen sources, such as fixation of atmospheric nitrogen [20] and in situ generation of ammonia from decomposition of urea in deep eutectic solvents [21]. Likewise, one-pot [2 + 2 + 1] procedures with *α*-haloketones **16**, 1,3-diketones **8** and primary amines catalyzed by heterogeneous acids have been developed [22,23]. Instead of *α-*haloketones, aryl glyoxals **16** (X = O) have been used for the synthesis of 3-hydroxy-substituted pyrroles **1** (R^3^ = OH) [24]. 4-Oxotetrahydrobenzofurans **18** are accessible from condensation of 1,4-diketones **17** and are transformed into their 4-oxotetrahydroindole analogs **1** with primary amines [25]. Next, addition of enehydroxyamines **19** onto alkynes **20** gives *O*-vinylhydroxylamines **21**, which can undergo a [3+3]-sigmatropic rearrangement followed by an intramolecular condensation to obtain the 4,5,6,7-tetrahydroindol-4-one structure **1** similar to a Trofimov reaction [26,27]. Furthermore, direct intramolecular and intermolecular metal-catalyzed cyclization between *β*-enaminone **22** and alkynes **20** has been reported [28,29] as well as a Cu-catalyzed 5-*exo*-dig annulation of alkyne-tethered enaminones [30]. Ceric (IV) ammonium nitrate (CAN) oxidative coupling of **22** with vinyl ethers **23** affords structural motif **1** and is an attractive strategy toward *N-*aryl 2,3-unsubstituted tetrahydroindol-4-ones [31]. Furthermore, azirines are interesting building blocks for pyrroles [32]. *2H*-Azirines **24** are prepared from isoxazoles **25** [33] or generated in situ from *α*-azidochalcones **26** [34]. The final example uses C-H insertion of *α*-imino rhodium carbenoids accessible from *N*-sulfonyl-1,2,3-triazoles **27** into 1,3-cyclohexanediones **8** [35].

## 3. Synthesis of [1,2]-Fused Polyheterocyclic Structures

### 3.1. Five-Membered Rings

#### 3.1.1. Electrophilic Aromatic Substitution

**Scheme 2:**
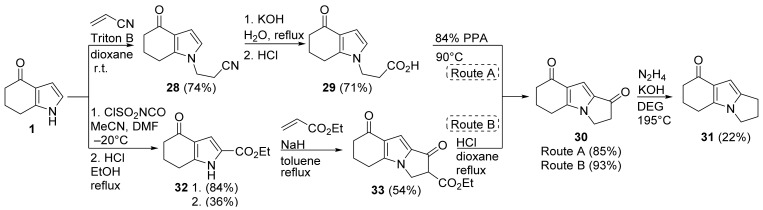
EAS onto pyrrole to obtain [1,2]-fused five-membered rings.

Electrophilic aromatic substitution (EAS) onto pyrroles readily occurs at the C-2 position. Sechi et al. *N*-alkylated pyrrole **1** with acrylonitrile to obtain **28** followed by basic hydrolysis to **9** and polyphosphoric acid (PPA) mediated intramolecular Friedel-Crafts acylation to afford polyheterocyclic structure **30** in an overall yield of 45% (Scheme 2 Route A) [36]. Interestingly, Wolff-Kishner reduction of **30** in diethylene glycol (DEG) was selective for one ketone functionality, leading to **31** in 22% yield. Later, an alternative, albeit less efficient route toward **30** was disclosed starting with an EAS on **1** with chlorosulfonyl isocyanate to introduce a 2-nitrile group which afforded ester **32** upon hydrolysis in 30% yield over two steps (Scheme 2 route B) [37]. *N*-Alkylation with ethyl acrylate followed by Dieckmann condensation to **33** and decarboxylation eventually produced tricycle **30** in an overall yield of 15%.

**Scheme 3:**
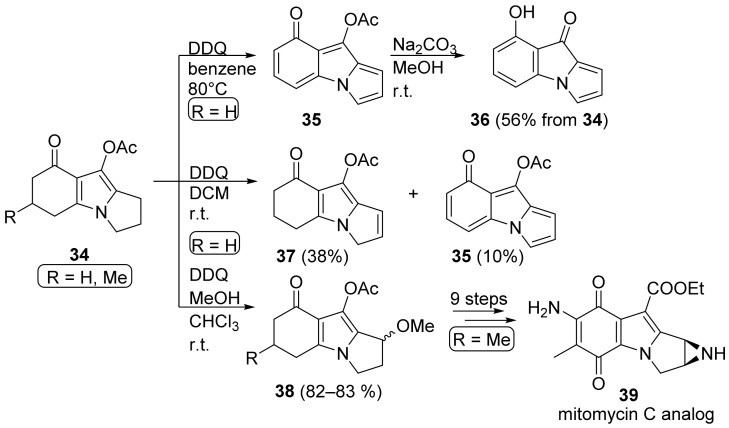
Oxidation of **34** under various conditions.

In 1995, Edstrom et al. examined the oxidation of similar structures (Scheme 3) [38]. **34** was previously synthesized using 1,3-cyclohexadiones **8** and proline derivatives of **15** according to the strategy depicted in Scheme 1 [17]. Treatment of **34** with excess of 2,3-dichloro-5,6-dicyano-1,4-benzoquinone (DDQ) in chloroform at 80 °C afforded **35**, which upon mild basic hydrolysis formed **36** in 56% yield over two steps. Limiting the amount of oxidant at room temperature and shorter reaction time provided a mixture of **37**, **35** and starting material **34**. Furthermore, by trapping the cationic intermediate with methanol, **38** was isolated which was used later on in the total synthesis of mitomycin C analog **39** in nine more steps [39].

**Scheme 4:**
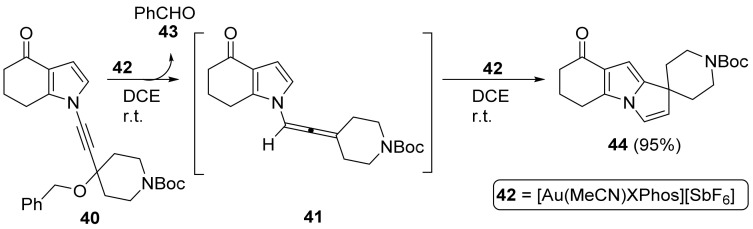
Synthesis of *N*-(allenyl)pyrrole **42** and subsequent EAS.

In 2017, Zhao et al. reported the synthesis of *N*-allenamides and pyrroles (Scheme 4) [40]. Under gold **42** catalysis, *N*-alkynylpyrrole bearing a benzyloxy group at the propargylic position **40** was converted into the corresponding allenepyrrole **41**, expelling benzaldehyde **43**. Next, the electrophilic gold species **42** activates the allene moiety for an intramolecular EAS on the C-2 of the pyrrole to obtain polycyclic spiro structure **44** in 95% yield.

#### 3.1.2. C-H Activation

**Scheme 5:**
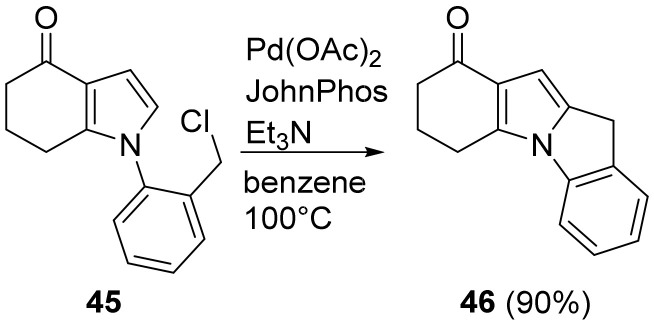
Tandem activation of benzyl halide and pyrrole **45** toward **46**.

Interest in C-H activation of pyrroles and indoles has increased over the years. This is due to the “atom economic” nature and the need for less reactive starting materials with a C-H bond instead of for example a C-X bond with X being a (pseudo)halide. Challenges are the inertness of the stronger C-H bonds compared to C-X bonds used in traditional metal-catalyzed cross-coupling reactions. Furthermore, the only small differences in C-H bond strengths lead to regioselectivity issues [41]. However, it should be noted that the electron rich nature of pyrrole already activates C-H bonds toward electrophilic metal species with the 2-position being more nucleophilic compared to the 3-position (as observed in classical EAS reactions due to better stabilization of the Wheland-type intermediate). Moreover, intramolecular reactions have less regioselectivity issues due to a limited degree of freedom. Chang et al. reported a Pd-catalyzed cyclization of *N*-(2-halobenzyl)-substituted 4,5,6,7-tetrahydro-4-indolone **45** which afforded a condensed pyrroloindole structure **46** via tandem activation of benzyl halide and the pyrrolic C-H bond (Scheme 5) [42].

**Scheme 6:**
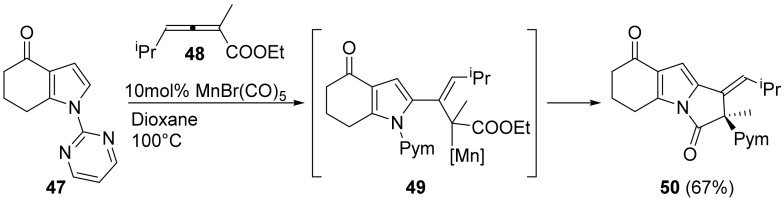
Mn^I^-catalyzed alkenylation of **47** and subsequent Smiles rearrangement.

For inactivated C-H bonds, a directing group (DG) is typically employed to ensure both reactivity and regioselectivity. A pyrimidine (Pym) group onto the *N*1-position of pyrrole **47** is often used to direct the transition metal to the C2-H bond. Wang et al. disclosed a Mn^I^-catalyzed alkenylation of *N*-DG indoles with allenes **48** (Scheme 6) [43]. Surprisingly, the strong nucleophilicity of the C-Mn bond leads to 1,4-migration of the directing group (Smiles rearrangement) with subsequent intramolecular substitution of the *N*-Mn onto the ester to stereoselectively afford a cyclized product. An extensive scope was made including various indoles, pyrroles, isoquinolin-1(*2H*)-ones and pyridine-2(*1H*)-ones as well as different directing groups and allenes with varying yields of 33–96%. In the context of this review, **47** was alkenylated with **48** to intermediate **49** followed by Smiles rearrangement and subsequent intramolecular substitution onto the ester, to provide **50** in 67% yield.

### 3.2. Six-Membered Rings

#### 3.2.1. Substitution

**Scheme 7:**
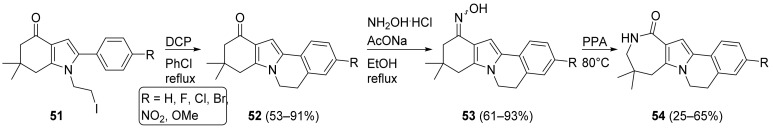
Intramolecular oxidative radical substitution and Beckmann rearrangement.

[1,2]-Annulated six-membered rings with the pyrrole moiety **52** have been synthesized via an intramolecular oxidative radical substitution of pyrroles bearing primary *N*-alkyl iodides **51** to aromatic carbon nuclei using dicumyl peroxide (DCP), acting both as radical initiator and oxidant (Scheme 7) [44]. Furthermore, Beckmann rearrangement on the oxime analogs **52** afforded conformationally restricted azepinepyrroloisoquinolinones **54** which structure can be found back in paullones with anticancer activity [45]. The conformationally restricted structures **54** were tested against various cancer cell lines, however, the antiproliferation activity was not improved compared to previous analogs [46].

**Scheme 8:**
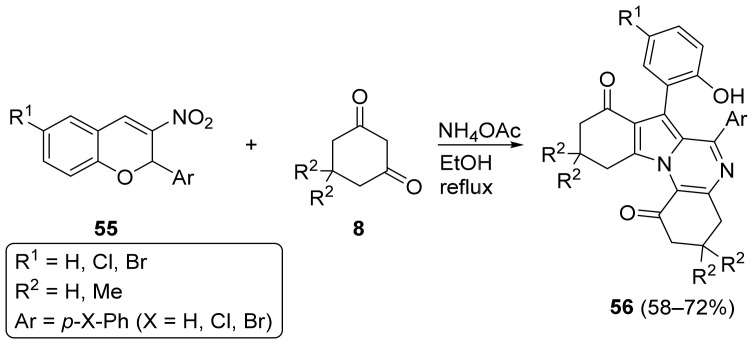
Three-component reaction toward **56**.

A three-component reaction was developed using various 1,3-cyclohexadiones **8**, 3-nitrochromenes **55**, and ammonium acetate which yielded functionalized 3,4,10,11-tetrahydroindolo[1,2-*a*]quinoxaline structures **56** in a cascade reaction forming the pyrrole and fused quinoxaline moiety in multiple steps, including two equivalents of cyclohexanedione in the final product (Scheme 8) [47,48]. A tentative reaction mechanism was proposed, although the exact mechanism is not known so far. Most likely, an oxidation step with HNO_2_ or oxygen is required. These complex polyheterocycles were obtained in 58–72% yield.

#### 3.2.2. C-H Activation

**Scheme 9:**
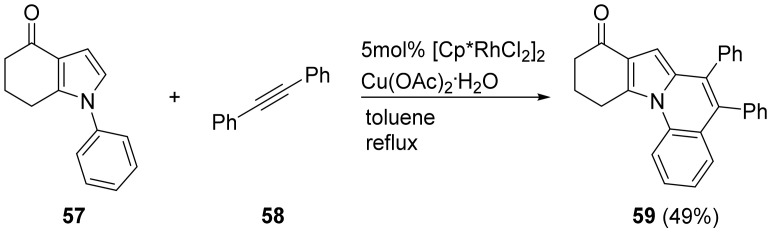
Rh(III)-catalyzed double C-H activation and alkyne annulation.

Chen et al. developed a Rh(III)-catalyzed double C-H activation of *α*-C-H bond of *N*-aryl azoles such as *N*-phenyl pyrroles without heteroatom directing assistance, followed by an annulation reaction with alkynes **58** to give pyrrolo-[*a*]fused quinolines (Scheme 9) [49]. A broad scope of *N*-(hetero)aryl and *N*-vinyl imidazoles provided the fused quinolines in good to excellent yields (43–99%). *N*-Aryl pyrroles gave lower, albeit acceptable yields (21–62%). When *N*-phenyl-4,5,6,7-tetrahydroindol-4-one **57** was used, **59** was obtained in 49% yield.

**Scheme 10:**
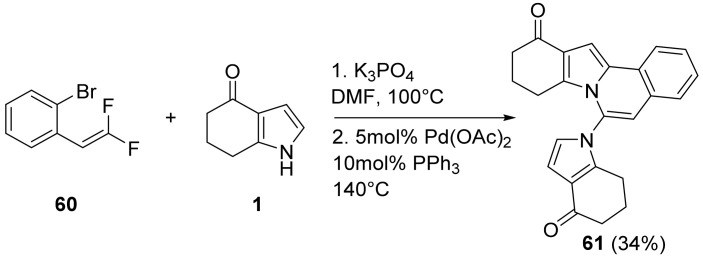
Synthesis of *N*-fused isoquinoline derivatives.

Tetrahydroindolo fused isoquinoline derivatives, having an additional tetrahydroindolone side group, were synthesized using a one-pot two-step reaction of 1-bromo-2-(2,2-difluorovinyl)benzenes with various N-H containing heterocycles including 4-oxotetrahydroindole **1** (Scheme 10) [50]. First, a double substitution of two fluorine atoms with the N-H heterocycles occurs, followed by a Pd-catalyzed intramolecular C-H arylation. With unsubstituted 2-(2,2-difluorovinyl)-1-bromo-benzene **60**, the yield of various indoles was high (71–84%) while electron poor N-H heterocycles gave lower yields (23–53%). Unsubstituted pyrrole and 4,5,6,7-tetrahydroindol-4-one **1** resulted in the envisioned product in 67% and 34%, respectively. This *N*-fused isoquinoline derivative **61** exhibits potent inhibitory activity for human nucleotide pyrophosphatase/phosphodiesterase 1 and 3 [50].

### 3.3. Seven-Membered Rings

#### Substitution

**Scheme 11:**
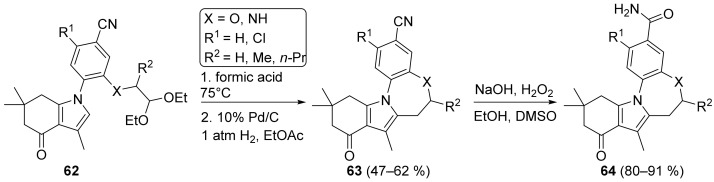
[1,2]-Annulated diazepine and oxazepine pyrrolocyclohexanone **64** synthesis.

[1,2]-Annulated diazepine and oxazepine pyrrolocyclohexanones **64** have been reported by Neubert et al. as potential Hsp90 inhibitors [51]. The introduction of a seven-membered ring was hypothesized to be entropically favorable to retain a dihedral angle of 47.4 degrees between the *N*-aryl and the pyrrole. Formation of these oxazepines and diazepines **64** was achieved in a multistep synthesis with the formation of the seven-membered ring, being an intramolecular EAS of an acetal onto the *α*-C of pyrroles **62** followed by catalytic reduction of the double bond (Scheme 11). The nitrile functionality of **63** was hydrolyzed to obtain the Hsp90 inhibitors. Activities of **64** were comparable with the freely rotatable *N*-aryl 4,5,6,7-tetrahydroindol-4-one, however, with improved calculated physical properties.

## 4. Synthesis of [2,3]-Fused Polyheterocyclic Structures

### 4.1. Five-Membered Rings

**Scheme 12:**
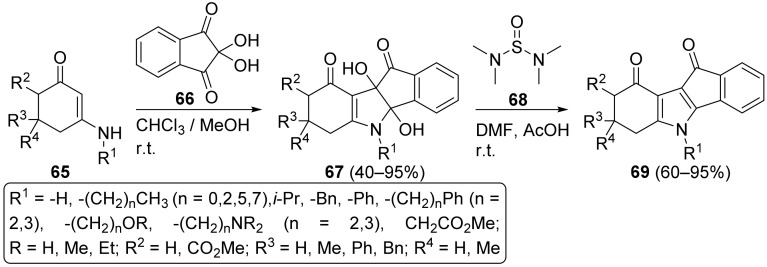
Synthesis of partially saturated indeno[1,2-*b*]indoles **69**.

Post-functionalizations of pyrrolocyclohexanones forming [2,3]-annulated five-membered rings are not reported. However, Hemmerling and Reiss disclosed a regioselective synthesis of *vic*-dihydroxy-indenoindoles **67** from 3-aminocyclohex-2-enones **65** and ninhydrin **66** (Scheme 12) [52]. Deoxygenation of **67** with *N,N,N’,N’*-tetramethylsulfurous diamide **68** provides an efficient and facile procedure for the synthesis of partially saturated indeno[1,2-b]indoles **69** derivatives which are a novel class of potent inhibitors of the human protein kinase CK2 and the breast cancer resistance protein ABCG2 [53,54,55,56].

### 4.2. Six-Membered Rings

#### 4.2.1. Rearrangement

**Scheme 13:**
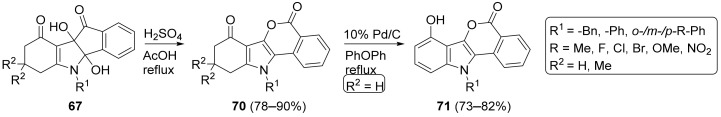
Synthesis and aromatization of isocoumarin fused pyrrolocyclohexanones **70**.

As an alternative to the deoxygenation of **67**, discussed in previous section, Pramanik et al. disclosed the acid-catalyzed rearrangement of **67** to isocoumarin fused pyrrolocyclohexanones **70** in good yields (78–90%) (Scheme 13) [57]. A mechanism is proposed where one alcohol forms a hydroxy epoxide intermediate with the adjacent ketone. Loss of water from the ortho-position of the nitrogen leads to the formation of a cationic intermediate followed by ring expansion through breaking the epoxy C-C bond to finally obtain **70**. Furthermore, dehydrogenation of **70** (R^2^ = H) with 10% Pd/C produced 4-hydroxyindole fused isocoumarins **71** in good yields (73–82%). These 4-hydroxyindole fused isocoumarins have interesting spectral properties, such as high quantum yields of fluorescence and ability to act as fluorescence “turn-off” sensor for Cu^2+^- and Fe^3+^-ions [57].

#### 4.2.2. Electrophilic Aromatic Substitutions

**Scheme 14:**
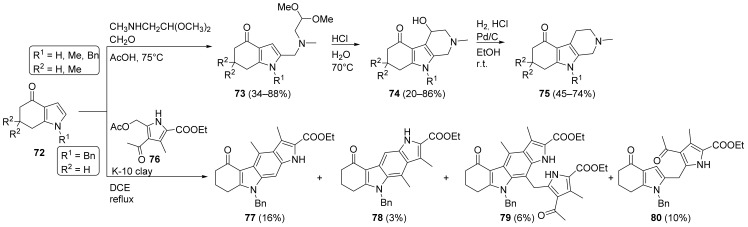
Double EAS on 2,3-unsubstituted 4,5,6,7-tetrahydroindol-4-ones **72**.

Mannich reaction with 2,3-unsubstituted 4,5,6,7-tetrahydroindol-4-one **72**, formaldehyde and aminoacetals affords acetal protected Mannich bases **73** which upon acidic hydrolysis ring-close to the corresponding hydroxy compounds **74** (Scheme 14). Finally, hydrogenolysis of **74** with Pd/C afforded various *β*-carboline derivatives **75** [11].

Dielectrophiles can react twice with pyrroles in a one-pot reaction in a similar fashion. Chunchatprasert and Shannon disclosed the double substitution of 2,3-unsubstituted pyrrolocyclohexanones **72** with pyrrole **76** (Scheme 14) [58]. *N-H* pyrrolocyclohexanone **72** was unreactive under the given conditions. However, with the *N*-benzyl derivative, **77** was obtained in 16% yield together with regioisomer **78** (3%). Moreover, 10-pyrrolylmethyl derivative **79** was obtained in 6% yield and acyl monosubstituted product **80** in 10% yield. The product **80** can be seen as a precursor to **77**.

**Scheme 15:**
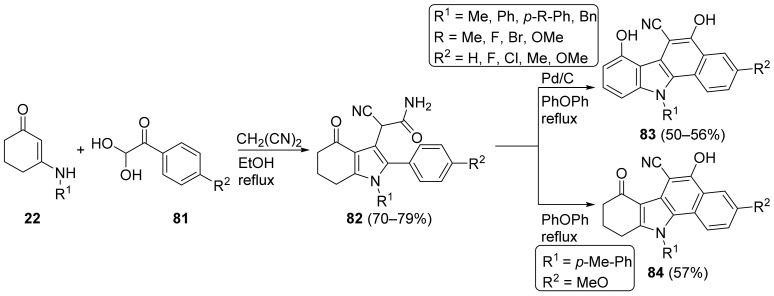
Thermal cyclization and Pd-catalyzed aromatization of **82**.

Pramanik et al. developed a multicomponent condensation reaction of enamines **22**, arylglyoxals **81** and malononitrile forming cyclohexanone-fused 2-(3-pyrrolyl)-2-cyanoacetamides **82** in high yields (70–79%) (Scheme 15) [59]. These 3-substituted 2-arylpyrrolocyclohexanones **82** are converted into highly substituted benzo[*a*]carbazoles **83** through a cyclization and Pd-catalyzed aromatization. Without Pd-catalyzed aromatization, **84** was isolated, which is evidence that the cyclization is indeed thermal.

#### 4.2.3. C-H Activation

**Scheme 16:**
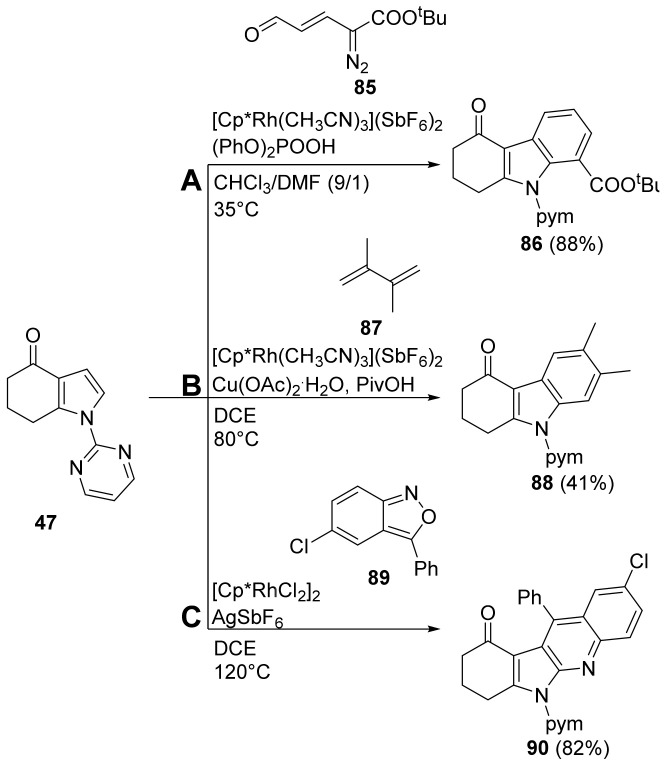
C-H activation of **47** toward [2,3]-benzo-fused and quinoline-fused 4,5,6,7-tetrahydroindol-4-one.

As was mentioned in Section 3.1.2, C-H activation on pyrroles is an attractive functionalization strategy. However, to overcome regioselectivity issues, a DG such as pyrimidine (pym) onto the *N*-pyrrole could direct the electrophilic metal-complex to formal C2-H activation. This strategy is used for the benzannulation of *N*-pyrimidine 4,5,6,7-tetrahydroindol-4-one **47** toward indolocyclohexanones. Wang et al. disclosed the Cp*Rh(III)-catalyzed benzannulation of pyrrolocyclohexanone **47** with enaldiazo compound **85** (Scheme 16A) [60]. A plausible mechanism was proposed wherein a rhodium carbenoid complex is formed from the enaldiazo compound **85**, followed by *α*-insertion and protonation which results in the alkenated intermediates. Brønsted acid-catalyzed double bond isomerization, followed by Friedel-Crafts cyclization and subsequent dehydration provides the benzo-fused product. The corresponding indole product **86** was obtained in 88% yield.

While this benzannulation is convenient, the availability of these enaldiazo compounds **85** is limited. Therefore, research has been done utilizing easily accessible, however, less reactive 1,3-dienes **87** as synthons for the benzannulation of *N*-heterocycles. Wang et al. described a Rh(III)-catalyzed formal oxidative [4 + 2] cycloaddition of nitrogen heterocycles, including **47**, with 1,3-dienes **87** (Scheme 16B) [61]. Mechanistically, the *π*-allyl metal species, which is generated via C-H activation and diene insertion, is trapped by the nucleophilic *β*-position of the *N*-heterocycles. Lastly, Cu(II)-catalyzed oxidation of the intermediate delivers the aromatized products. Due to the electron withdrawing effect of the ketone moiety in **47** which limits the nucleophilicity of the *β*-position, **88** was obtained in only 41% yield.

The disrupted aromaticity and weak N-O bond causes anthranils (2,1-benzisoxazoles) like **89** to be unstable, though interesting building blocks in organic chemistry. Kim et al. developed a regioselective Cp*Rh(III)-catalyzed direct amination of **47** with **89** followed by an intramolecular cyclization to afford **90** in 82% yield (Scheme 16C) [62]. Mechanistically, a cationic Rh(III) complex can be generated with the pyrrole via a formal C-H activation process. Coordination of the anthranil and migratory insertion provides the amino species which upon protonation delivers the C-2-aminated pyrrole which can further cyclize with the formed ketone to **90**.

**Scheme 17:**
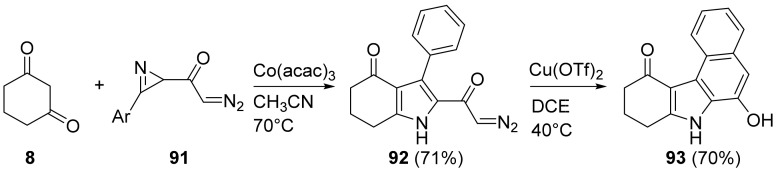
Metal-catalyzed intramolecular aromatic substitution onto an *α*-diazocarbonyl moiety.

Similar to the intermolecular aromatic substitution of Rh-carbenoid complexes with C2-H activated pyrroles, Khlebnikov et al. disclosed the intramolecular aromatic substitution of 3-phenyl-2-(diazoacetyl)-tetrahydroindolone **92** which was formed from 2-diazoacetylazirines **91** and 1,3-cyclohexadione **8** (Scheme 17) [63]. Interestingly, use of the Co(III) catalyst did not affect the diazoacetyl functionality. Next, a Cu(OTf)_2_ catalyzed intramolecular aromatic substitution of the 3-aryl group onto the copper carbenoid delivered interesting benzo-fused 1*H*-indol-7-ol **93** in 70% yield.

## 5. Synthesis of [3,4]-Fused Polyheterocyclic Structures

### 5.1. Five-Membered Rings

**Scheme 18:**
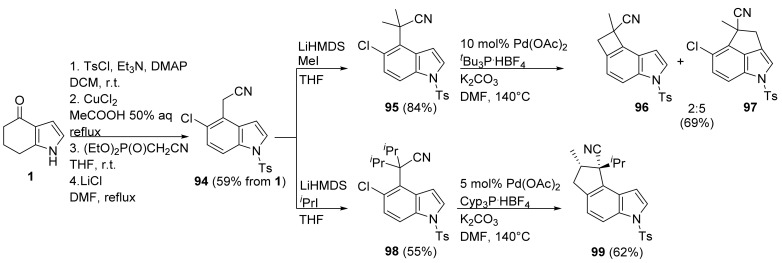
Intramolecular Pd-catalyzed alkane C-H arylation from aryl chlorides.

Only one example that uses the pyrrolocyclohexanone structure for the synthesis of [3,4]-annulated five-membered rings can be found in literature. This is most likely due to the considerable ring strain of these structures. Baudoin et al. reported the intramolecular Pd-catalyzed alkane C-H arylation from aryl chlorides [64]. One example of the extensive scope uses **94** which is synthesized starting from 4,5,6,7-tetrahydroindol-4-one **1** via tosylation (83% yield) and double chlorination with CuCl_2_ (83% yield), followed by a Horner-Wadsworth-Emmons reaction (quantitative) and aromatization with loss of one chloride (85%) (Scheme 18). Double methylation of **94** delivered **95**. Intramolecular Pd-catalyzed methyl C-H arylation of **95** afforded the expected cyclobutarene **96** surprisingly together with **97** as an inseparable mixture with a ratio of 2:5, respectively. The unintended isomer **97** presumably arises from a palladium migration, however, the exact mechanism is not elucidated. The same procedure was used for the synthesis of indane **99** starting from **98** with only one diastereomer being observed as a result of steric hindrance.

### 5.2. Six-Membered Rings

#### 5.2.1. Condensation Reactions

**Scheme 19:**
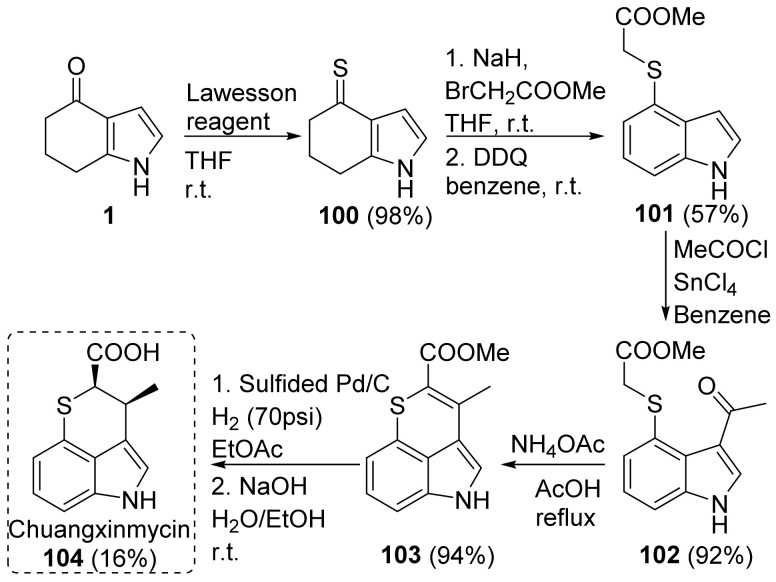
Total synthesis of Chuangxinmycin **104**.

Due to the antitumoral and antibiotic activity of Chuangxinmycin **104**, synthesis of analogs of this natural product have gained research interest. Murase et al. reported the synthesis of 4-alkylsulfanylindole **101** starting from **1** by converting the ketone to thioketone **100**, followed by alkylation with methyl bromoacetate and DDQ-mediated dehydrogenation [65]. The sulfide **101** is an interesting building block for the [3,4]-fused indole core. Kozikowski et al. acetylated **101** at the 3-position, followed by intramolecular Knoevenagel condensation of **102** to obtain dehydrochuangxinmycin methyl ester **103** [66]. Reduction of the double bond with hydrogen gas and a sulfided Pd/C catalyst, followed by hydrolysis of the ester afforded Chuangxinmycin **104** (Scheme 19).

**Scheme 20:**
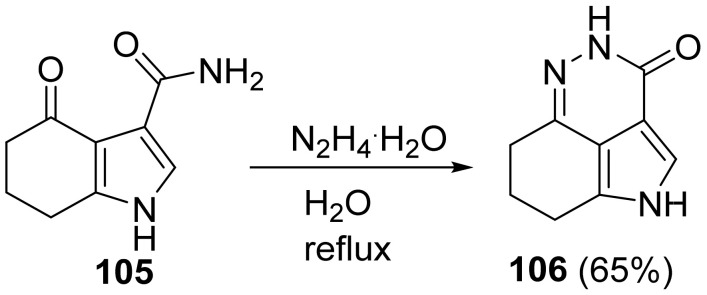
Formation of a tricyclic pyrrolo fused cinnolinone.

1,4-Dicarbonyls can condense with hydrazine to give access to six-membered rings. Thus, treatment of 4-oxo-4,5,6,7-tetrahydroindole-3-carboxamide **105** with hydrazine formed tricyclic pyrrolo fused cinnolinone **106** in 65% yield (Scheme 20) [67]. Similar compounds have been tested against several tumor cell lines, however, only low tumor growth cell inhibitory activities were observed [68].

**Scheme 21:**
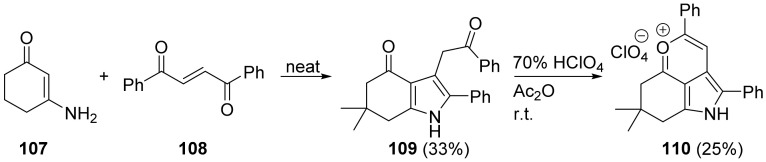
Synthesis of pyrrole fused pyrylium salt **109**.

Pyrrolo fused pyrylium salts are useful synthetic precursors of the corresponding pyrrolo fused pyridines via ring opening and recyclization with ammonium or hydrazine acetate [69]. Pyrylium salts are accessible by dehydration of 1,5-dicarbonyls with an acylating mixture of 70% perchloric acid and acetic anhydride (Scheme 21). These 1,5-dicarbonyls, e.g., **109** are obtained by condensing various *β*-enaminones such as **107** with dibenzoylethylene **108**. The pyrylium salts were obtained in 91–98% yield with the exception of tetrahydroindole derivative **109** with only 25% yield of **110** due to considerable ring strain. Furthermore, in the same report various pyrylium salts were converted to the corresponding pyridines, pyridinium salts or pyridinones. However, no conversion of **109** to any corresponding *N*-containing six-membered ring was reported. Probably, this conversion did not succeed because of the ring strain of this system.

#### 5.2.2. C-H Activation

**Scheme 22:**
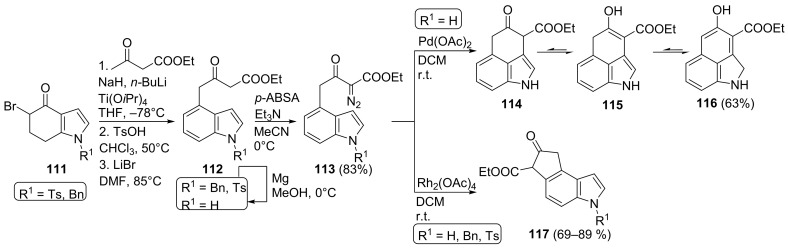
Formal C-H insertion of metal carbenoids in the C3-H or C5-H bond.

The tetrahydrobenzo[*cd*]indole **115** has an interesting skeleton which can be found in a variety of natural products, e.g., in ergoline alkaloids. Formal intramolecular C-H bond insertion by diazoketones has become an attractive strategy toward this constrained skeleton. Thus, Matsumoto and Watanabe disclosed the synthesis of 4-(4-indolyl)-3-oxobutanoic acid derivatives **113** (Scheme 22) [70]. Introduction of a 5-halogen substituent onto the tetrahydroindolone, utilizing CuX_2_ such as in compound **111** ensures mild aromatization later on in the synthesis. Condensation of ketone **111** with double anionic ethyl acetoacetate followed by LiBr mediated aromatization yields **112**. Furthermore, the ketoesters **112** were converted to the diazo compounds **113** with 4-acetamidobenzenesulfonyl azide (*p*-ABSA) which can undergo a metal-catalyzed formal intramolecular C-H bond insertion. Matsumoto, Watanabe and Kobayashi reported the use of Pd(OAc)_2_ as the catalyst for the synthesis of structure **114** [71]. However, while using Rh_2_(OAc)_4_, as well as Cu(acac)_2_, the C-H insertion occurred on the 5-position to obtain a [4,5]-annulated five-membered structure **117**. It is worth mentioning that *N*-substituted indoles did not form tetrahydrobenzo[*cd*]indole **114**. However, under rhodium catalyzed conditions, *N*-substituents were compatible and **117** was formed in good yields. Hansen et al. further investigated the introduction of the diazo functionality at an earlier stage in the synthesis, however, they concluded that the diazo is best introduced at the end [72]. Additionally, Rosenberg reported that **114** was isolated in equilibrium with its tautomer **115** which could undergo a proton transfer to form the thermodynamically preferred naphthalene derivative **116** (Scheme 22).

### 5.3. Seven-Membered Rings

**Scheme 23:**
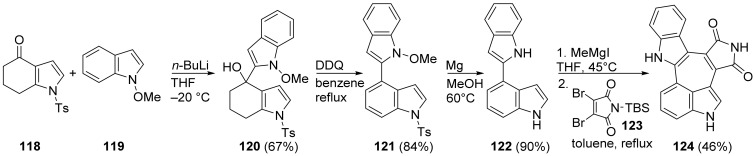
Total synthesis of arcyriacyanin A **124**.

Arcyriacyanin A **124** is a green-blue pigment found in the mycetozoa *Arcyria obvelata* with potential anticancer activity. It was synthesized by Murase et al. by nucleophilic addition of lithiated 1-methoxyindole **119** on the ketone functionality of *N*-tosyl 4,5,6,7-tetrahydroindol-4-one **118** to give **120**, which was treated with DDQ to obtain the unsymmetrical 2,4′-biindole **121** (Scheme 23) [73]. Deprotection of both the indoles was accomplished by reaction with magnesium in methanol. Finally, **122** can be transformed by formation of the bismagnesium salt followed by double substitution onto *N*-*tert*-butyldimethylsilyl (TBS) dibromomaleimide **123** which is deprotected under the reaction conditions. Arcyriacyanin A **123** was evaluated as cell growth inhibitor against a panel of human cell lines, however, the EC_50_ value was rather high [73].

## 6. Synthesis of [4,5]-Fused Polyheterocyclic Structures

### 6.1. Five-Membered Rings

#### 6.1.1. Hantsch Thiazole Synthesis

**Scheme 24:**
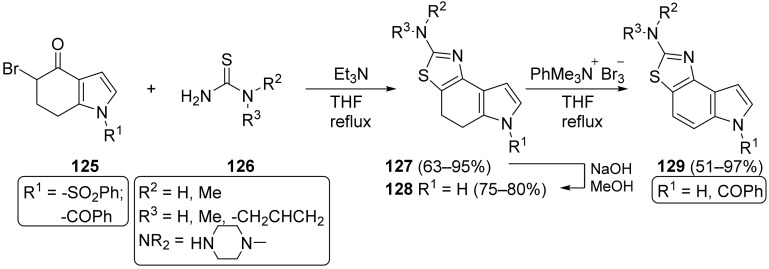
Aminothiazole fused indole synthesis.

Condensation of *α*-bromoketones with thiourea remains one of the most reliable routes to aminothiazoles. Remers et al. condensed *N*-protected benzenesulfonyl and benzoyl 5-bromo-4-oxo-4,5,6,7-tetrahydroindoles **125** with substituted thioureas **126** (Scheme 24) [67,74]. Surprisingly, in ethanol with an excess of triethylamine, exclusively the imine is formed without substitution of the bromine. However, when the reaction is executed in methanol, all tetrahydroindoles are converted to the corresponding thiazole **127**. The benzenesulfonyl-group as well as the benzoyl-group can be deprotected with NaOH in methanol. DDQ mediated dehydrogenation of unprotected **128** furnished the fully aromatic derivatives **129** in low yield. DDQ mediated aromatization of benzoyl-protected derivatives **127** was unsuccessful. However, phenyltrimethylammonium tribromide has proven to be very effective for dehydrogenation of *N-H* **128** and *N*-benzoyl **127**. Interestingly, **128** with R^2^ and R^3^ being a methyl or NR_2_ being a piperazine moiety, showed anti-inflammatory activity while only low activities were found for in vitro antibacterial and antifungal screenings [67].

#### 6.1.2. [4 + 1] Paal-Knorr Type Syntheses from 1,4-Diketones

**Scheme 25:**
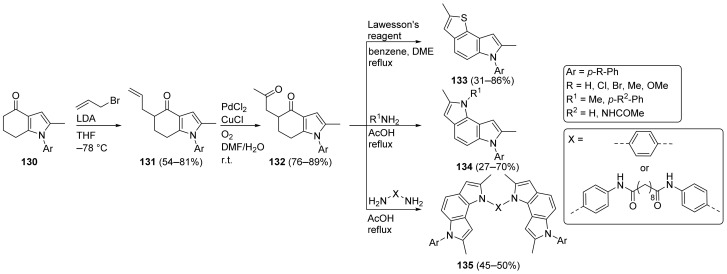
Paal-Knorr syntheses toward thieno- and pyrrolo-indoles as potential DNA intercalators.

Synthesis of [4,5]-fused five-membered heterocycles can be realized using the Paal-Knorr condensation of 1,4-dicarbonyls with nucleophiles. Alkylation of *N*-substituted 4,5,6,7-tetrahydroindol-4-one **130** with chloroacetone did not afford the desired 1,4-diketone building blocks **132**. Therefore, Martínez and Oloarte reported the allylation of **130** with allyl bromide using lithium diisopropylamide (LDA) to obtain **131** followed by a Wacker-Tsuji oxidation (Scheme 25) [75]. Furthermore, **132** was condensed with Lawesson’s reagent in a benzene-dimethoxyethane (DME) mixture or methylamine hydrochloride, and aromatization occurred under the reaction circumstances to obtain thieno-**133** and pyrrolo-indoles **134**, respectively. Interestingly, the aromatization step occurred without additional oxidant. Later on, Chacón-García and Martínez reported the cytotoxic properties of these pyrrolo-**134** and thieno-indoles **133**, most likely due to DNA intercalation [76]. Various anilines as well as dianilines were used to build a small library of potential DNA intercalators **133** and **134**, and double DNA intercalators **135**, respectively.

#### 6.1.3. [3 + 2 ] Paal-Knorr Type Syntheses from 1,3-Dicarbonyls

**Scheme 26:**
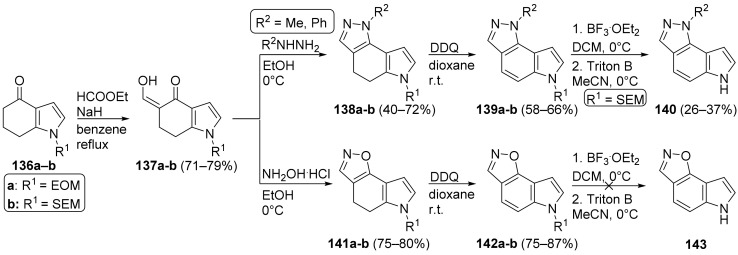
Synthetic pathway toward tricyclic indoles from 1,3-dicarbonyls.

Similar as in the Paal-Knorr pyrrole and thiophene synthesis, five-membered heterocycles can be synthesized from 1,3-dicarbonyls and 1,2-dinucleophiles such as hydrazine and hydroxylamine to obtain pyrazoles and isoxazoles, respectively. Inspired by work of Remers and coworkers [74], Nikolaropoulos et al. reported the formylation of ethoxymethyl (EOM) protected 4,5,6,7-tetrahydroindol-4-one **136a** with ethyl formate in the presence of sodium hydride to obtain enol tautomer **137a** (Scheme 26) [77]. Condensation with methylhydrazine or phenyl hydrazine afforded the corresponding pyrazole **138a** (R^1^ = EOM) regioselectively in 72% and 46% yield, respectively. Similarly, condensation of **137a** with hydroxylamine hydrochloride provided the isoxazole fused dihydroindole **141a** in 75% yield. Subsequently, the derivatives **138a** and **141a** were dehydrogenated on treatment with DDQ in dioxane to the corresponding fused tricyclic indole derivatives **139a** (R^2^ = Me, Ph) and **142a**, respectively. However, extensive attempts to cleave the EOM group of **139a** and **142a** with HCl did not yield the *N*-deprotected derivatives **140** or **143**, respectively. When a 2-(trimethylsilyl)ethoxymethyl (SEM) group is used instead of EOM, deprotection of the pyrazole fused tricyclic indole derivatives **139b** afforded the *N*-unprotected products **140** in 26% (R^2^ = Me) and 37% (R^2^ = Ph) yield, respectively. However, deprotection of the isoxazole-fused tricyclic indole **142b** led to decomposition. Evaluation of the inhibitory activity of these fused tricyclic indoles against soluble guanylate cyclase (sGC) demonstrated in vitro activity with **142a** being the most potent derivative [77].

**Scheme 27:**
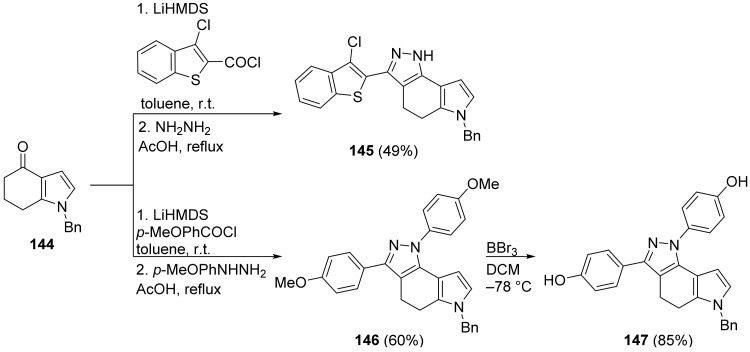
One-pot procedure toward pyrazole-fused dihydroindoles.

In a study concerning the cytostatic activity in cancer cell lines of novel conformationally rigid pyrazoles, Kasiotis et al. synthesized two pyrazole-fused tetrahydroindol-4-ones [78]. Therefore, benzyl-protected tetrahydroindol-4-one **144** in the presence of lithium hexamethyldisilazane (LiHMDS) base under anhydrous conditions provided the corresponding enolate, which was subsequently condensed with 3-chlorobenzothiophene-2-carbonyl chloride or *p*-anisoyl chloride to afford the 1,3-diketone derivatives (Scheme 27). The one-pot condensation of the latter with hydrazine and its methoxyphenyl counterpart afforded the desired pyrazole derivatives **145** and **146** in 49% and 60% yield, respectively. Demethylation of **146** delivered diphenolic compound **147** in 85% yield. Evaluation of the cytostatic activity revealed that **147** had the most potent inhibitory activity against the growth of the panel of tested cancer cell lines [78].

#### 6.1.4. [3,3]-Sigmatropic Rearrangements

**Scheme 28:**
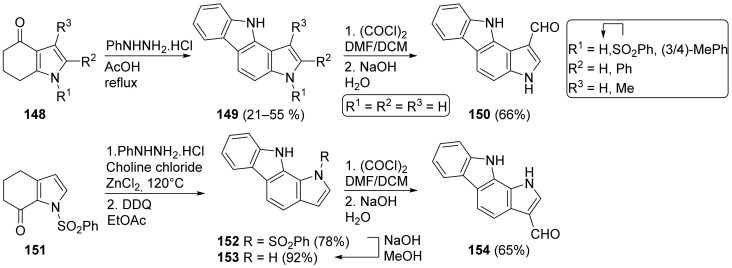
Fischer indole synthesis.

The ketone functionality in 4-oxotetrahydroindole **148** can be converted to arylhydrazones that can be subjected to [3,3]-sigmatropic rearrangements leading to the Fischer indole synthesis. Condensing phenylhydrazine hydrochloride with **148** in acetic acid and subsequent rearrangement gave the pyrrolo[3,2-*a*]carbazoles **149** in yields of 21–55% (Scheme 28) [79]. Surprisingly, aromatization occurred without additional oxidant. Furthermore, the reaction did not occur when two methyl groups were placed on the 6-position of the tetrahydroindolone **148**, most likely due to steric hindrance. In subsequent work, these pyrrolocarbazoles **149** were investigated for their selective kinase inhibitory activity in solid cancer treatment (Scheme 28) [80]. However, Fischer indole synthesis with unsubstituted tetrahydroindolone **1** resulted in poor yields and purification difficulties. When benzenesulfonyl-protected 4,5,6,7-tetrahydroindol-4-one **148** (R^1^ = SO_2_Ph, R^2^ = R^3^ = H) was used, the corresponding pyrrolo[3,2-*a*]carbazole **149** was isolated in 24% yield. Smooth deprotection and formylation afforded **150** as a potential kinase inhibitor. However, the inhibitory activity of the isomeric pyrrolo[2,3-*a*]carbazole **154** which was prepared similarly starting from 4,5,6,7-tetrahydroindol-7-one **151** was superior to **150**. Changing the solvent for the Fischer indole synthesis from acetic acid to a choline chloride–zinc chloride ionic liquid with subsequent one-pot DDQ oxidation increased the yield of **152** from 17% to 78% [80].

**Scheme 29:**
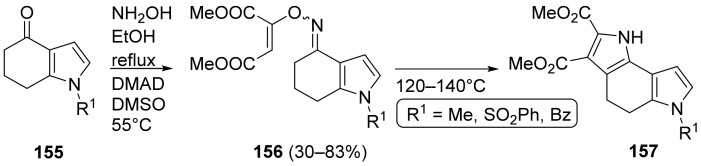
[3+3] sigmatropic rearrangement of *O*-alkenoates.

Instead of indole formation, a related thermal [3,3] sigmatropic rearrangement of *O*-alkenoates gives rise to a pyrrole-fused pyrrolocyclohexanone structure (Scheme 29) [81]. *O*-Alkenoates of 4,5,6,7-tetrahydroindol-4-one were synthesized by condensing hydroxyl amine with **155** followed by addition onto dimethyl acetylenedicarboxylate (DMAD) to obtain **156**. Thermal rearrangement at 120–140 °C afforded the corresponding pyrrole-fused tetrahydroindoles **157** in low yields (exact yield was not reported). Interestingly, oxidation to the benzodipyrrole did not occur at these elevated temperatures. Thermal rearrangement of *O*-vinylketoximes of **156** failed due to a retro-Michael addition.

#### 6.1.5. 1,3-Dipolar Cycloaddition

**Scheme 30:**
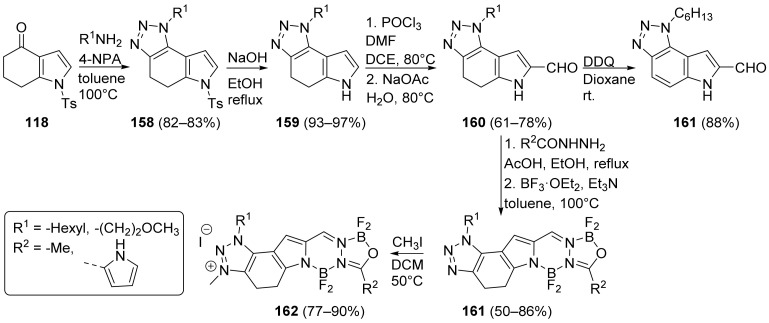
Synthesis of 1,2,3-triazole-linked BOPAHY dyes and their triazolium salts.

Recently, our group and Hao’s group independently introduced bis(difluoroboron) pyrrole acylhydrazones (BOPAHY) [82,83]. This novel fluorophore shows intriguing photophysical properties, including high fluorescent quantum yields in solution and in solid state and tunable absorption/emission properties. Very recently, our group reported the synthesis and spectroscopic properties of novel 1,2,3-triazole BOPAHY dyes and their corresponding triazolium salts (Scheme 30) [84]. Thus, we started from *N*-tosyl-4,5,6,7-tetrahydroindol-4-one **118** and performed our in-house developed general metal-free triazolization reaction [85,86,87,88,89]. This route toward 1,2,3-triazoles starts from enolizable ketones, primary amines and 4-nitrophenyl azide as diazo-transfer agent. The reaction of *N*-tosyl-4,5,6,7-tetrahydroindol-4-one **118** with hexylamine or 2-methoxyethyl amine afforded the corresponding 1,2,3-triazole **158** in 82% and 83%, respectively. It is worth mentioning that without tosyl protection the triazolization reaction did not occur. Deprotection of the tosyl group with NaOH resulted in the free *NH*-pyrrole **159** which could be *α*-formylated with a Vilsmeier reaction. These pyrrole-2-carbaldehydes **160** were then used in the synthesis of BOPAHY dyes **161** by condensation with acyl hydrazides and subsequent complexation with BF_3_·OEt_2_ in a one-pot procedure. Furthermore, oxidation of **160** with DDQ delivered the tricyclic 1,2,3-triazolo fused indole **161**. The obtained 1,2,3-triazole BOPAHYs were methylated with to obtain 1,2,3-triazolium BOPAHY salts **162** with limited water solubility.

### 6.2. Six-Membered Rings

#### 6.2.1. [4 + 2]-Cycloadditions

**Scheme 31:**
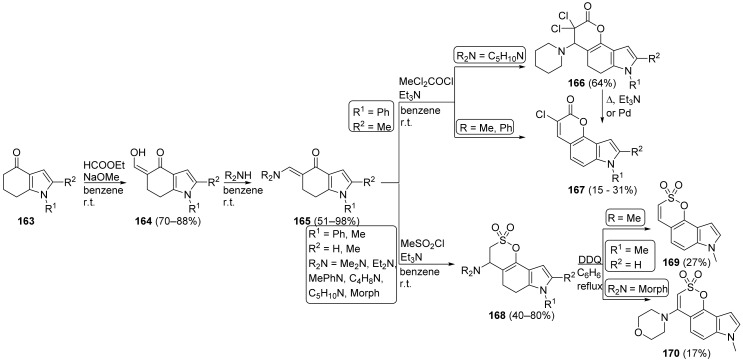
[4 + 2] cycloaddition to afford 4,5-fused six-membered rings.

4,5-Fused six-membered rings on the indole nucleus are interesting as this may lead to potentially pharmacologically active compounds. Therefore, the 4,5,6,7-tetrahydroindol-4-one is particularly suitable for the synthesis of these tricyclic heterocycles. Reaction of 4,5,6,7-tetrahydroindol-4-ones **163** with ethyl formate and sodium methoxide or potassium *tert*-butoxide regioselectively *α*-formylated the ketone functionality to **164** in 70–88% yield (Scheme 31) [90]. Enaminoketones **165** were prepared from **164** and secondary amines in 51–98% yield. These enaminoketones **165** are the starting products for multiple tricyclic heterocycles. [4 + 2]-Cycloaddition of **165** with dichloroketene (generated in situ from reaction of dichloroacetyl chloride and triethylamine) only resulted in the expected [4 + 2]-cycloaddition product **166** with R-N-R being a piperidino group. However, the other examples all afforded compound **167**. [4 + 2]-Cycloaddition of **165** with sulfene (generated in situ from elimination reaction of mesyl chloride with triethylamine) occurred readily in the case of R being aliphatic to give 1,2-oxathiino 2,2-dioxide fused 6,7-dihydrohydroindole structure **168** [91]. Complete aromatization of **168** was tried with DDQ. When R-N-R was a dimethylamino group, elimination of the amino group and aromatization occurred to obtain the least substituted 1,2-oxathiino[6,5-*e*]indole **169** in low yield. However, with R-N-R being morpholine, aromatization occurred without loss of the amino group to obtain **170**.

#### 6.2.2. Multistep Condensation Reactions

**Scheme 32:**
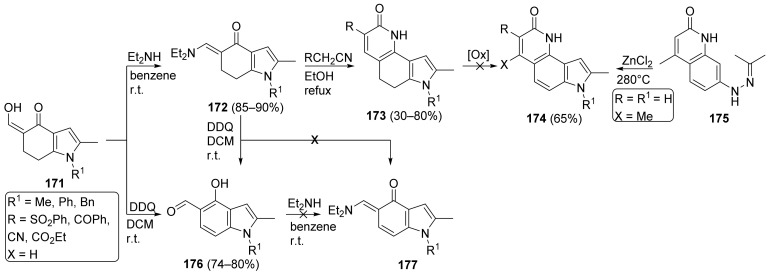
Synthesis of 5,6-dihydropyrrolo[2,3-h]quinolinones **168** and attempted aromatization.

Dall’Acqua et al. reported the synthesis of 5,6-dihydropyrrolo[2,3-*h*]quinolinones **174** as possible DNA intercalators with photo-binding ability and with lower toxicity compared to psoralens used in photo treatment of several skin diseases by intercalation and [2 + 2] photocycloaddition with DNA strands (Scheme 32) [92]. *β*-Aminoenones **172** discussed in the previous section are useful building blocks for this synthesis by conjugate addition of various cyanomethylene (RCH_2_CN) compounds followed by elimination of Et_2_NH. Next, cyclization to a non-isolable *2H*-pyran-2-imine intermediate followed by a Dimroth-type rearrangement affords 5,6-dihydropyrrolo[2,3-*h*]quinolinones **173** [93]. In order to obtain flat molecules which could intercalate in double stranded DNA, similar to the natural product angelicin, aromatization of **173** was attempted, however, it did not succeed due to solubility issues [94]. Furthermore, oxidation of the *β*-aminoenones **172** did only afford traces of the oxidized hydrolyzed product **179** which could be obtained directly from **171** in good yields. Attempts to synthesize the oxidized *β*-aminoenones **177** from **176** was unsuccessful. Therefore, an alternative synthetic pathway was followed for the synthesis of pyrrolo[2,3-*h*]quinolinone **174** analogs where the pyrrole ring is formed with a Fischer indole synthesis starting from hydrazone **175**. Surprisingly, **174** did not inhibit proliferation of tumor cell lines in phototherapy while the 5,6-dihydropyrrolo[2,3-*h*]quinolinones **173** bearing a phenylsulfonyl group exhibited high photoactivities [94]. Regarding the mechanism of action, **173** did not intercalate with DNA upon irradiation which is of great relevance in decreasing long-term toxic effects such as mutagenesis. It seems that lysosomes and/or mitochondria could be targeted by **173** induced photodamage to lipids and proteins with the involvement of free radicals.

**Scheme 33:**
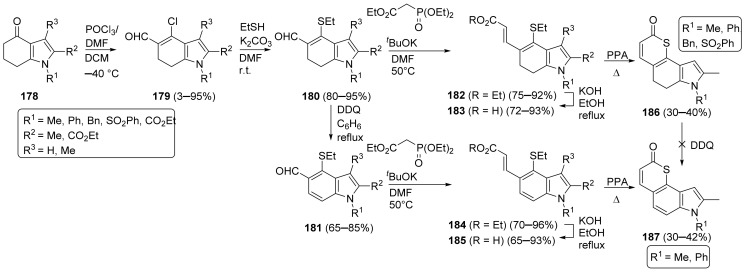
Synthesis of thiopyrano[2,3-*e*]indol-2-ones.

In 2005, Dall’Acqua et al. reported the synthesis of thiopyrano[2,3-*e*]indol-2-ones **187**, resembling angelicin analogs with potential photochemotherapeutic activities [95]. Due to the interesting results, further research on this work was done and an extended report was published in 2008 (Scheme 33) [96]. Chloroformylation of various 4,5,6,7-tetrahydroindol-4-ones **178** with the Vilsmeier–Haack reagent provided unstable aldehyde **179**. Low yields were obtained with *N*-unsubstituted tetrahydroindol-4-ones due to solubility issues. When the pyrrole moiety was not fully substituted, regioselectivity and overformylation issues occurred due to pyrrole formylation. Nucleophilic substitution of the chlorine atom with ethanethiol afforded thioethers **180**. Oxidation of these thioethers with DDQ afforded the corresponding stable aromatic aldehydes **181** in good yields. Wittig–Horner reaction onto aldehydes **180** and **181** afforded **182** and **184**, respectively, in good yields. Hydrolysis of the ester provided carboxylic acids **183** and **185**. Polyphosphoric acid (PPA) catalyzed cyclization succeeded only in low yield (30–40%) for the dihydrothiopyrano indoles **186** except with R^2^ bearing an ester or acid functionality. In the case of the aromatized acids **185**, only two examples afforded the corresponding thiopyranoindoles **187**. Direct oxidation of **186** was not successful. All derivatives of **186** and **187** possessed photo-antiproliferative activity with the most active being the fully aromatic compound **187** with R^1^ = Me [95]. In contrast with the series **173** and **174**, the dihydro derivatives **186** were less active. However, analogous to **173**, these compounds were not able to intercalate and form covalent adducts with DNA upon UV irradiation, although they were able to photo oxidize DNA bases, in particular pyrimidine bases. Again the involvement of free radicals, in particular the hydroxyl radical, was proven [96]. It was again postulated that mitochondria and liposomes are targeted, inducing apoptosis.

**Scheme 34:**
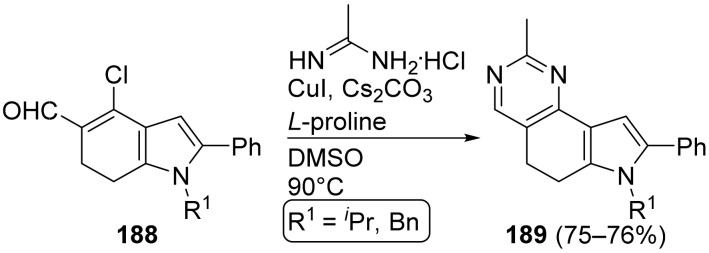
Synthesis of pyrroloquinazolines.

The chloroformylated product **188** can condense with 1,3-dinucleophiles. Batra et al. reported the copper-mediated coupling of **188** with acetamidine hydrochloride to obtain pyrroloquinazolines **189** in good yields (75–76%) (Scheme 34) [97]. No oxidation attempts of **189** have been reported.

**Scheme 35:**
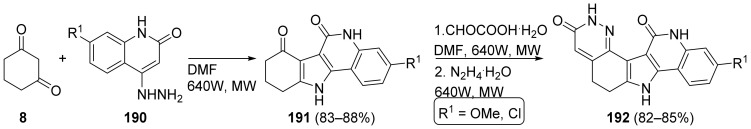
Microwave assisted synthesis of a pentacyclic ring system.

The indolo[3,2-*c*]quinolinone and pyridazinoquinoline structures are found in naturally occurring alkaloids and are extensively studied in medicinal chemistry. Due to the biological relevance of the indole, quinoline and pyridazine nucleus, Dandia et al. combined these moieties to obtain a pentacyclic heterocyclic ring system **192** (Scheme 35) [98]. Therefore, the tetrahydroindolone-fused quinolinone structure **186** was prepared via a Fischer indole cyclization of (2-oxo-1,2-dihydroquinolin-4-yl)hydrazine **185** and 1,3-cyclohexadione **8** under microwave irradiation with a small amount of DMF as energy transfer and dehydrating agent. One-pot reaction of key intermediate **186** with glyoxalic acid monohydrate with a few drops of DMF under microwave conditions followed by addition of hydrazine hydrate afforded **187** after 3–5 min in 85–88% yield without the need for further purification.

**Scheme 36:**
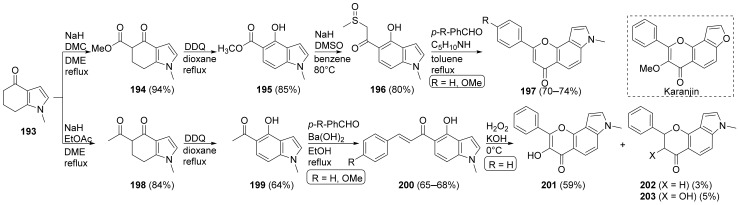
Synthesis of pyrroloflavanoids as Karanjin derivatives.

Furanoflavonoids such as karanjin are naturally occurring compounds which possess various kinds of biological activities. Maurya et al. explored the potential of the furanoflavonoid nucleus as antifungal and antibacterial agents and synthesized pyrrole and thiophene analogs of furanoflavonoids [99]. For the pyrrole analogs, 1-methyl 4,5,6,7-tetrahydroindol-4-one **193** was acylated with dimethyl carbonate (DMC) or ethyl acetate (EtOAc) with NaH as a base, which resulted in methyl 5-carboxylate **194** and 5-acetyltetrahydroindolone **198**, respectively (Scheme 36). Thereafter, DDQ mediated dehydrogenation of **194** and **198** afforded indoles **195** and **199**. Nucleophilic acyl substitution of the dimsyl anion on ester **195** afforded *β*-ketosulfoxide **196**, which upon treatment with substituted benzaldehydes and piperidine in toluene produced pyrroloflavone analogs **197** in 70–74% yield. Chalcone **200** was achieved in good yields via a Claisen–Schmidt condensation of **199** with benzaldehydes in the presence of barium hydroxide. Flavanol **201** was achieved applying an Algar-Flynn-Oyamada reaction of the corresponding chalcone **200** (R = H) in 59% yield together with two minor products **202** and **203**. When R = OMe, a complex reaction mixture was obtained. Screening **195**, **197**, **198**, **200** and **201** together with the thiophene analogs against various bacteria and fungi demonstrated higher inhibition activities of thiophene analogs compared to the pyrrole analogs [99]. Nevertheless, chalcone **200** (R = H) and flavone **197** (R = H) showed minimum inhibitory concentrations (MIC) against fungi comparable with natural furanoflavonoid karanjin.

**Scheme 37:**
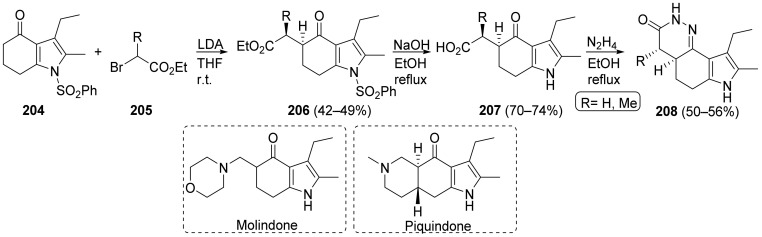
Synthesis of pyridazin-3-(*2H*)-one fused dihydroindoles **203**.

In search of analogs of the well-established antipsychotics Molindone and Piquindone, *N*-benzenesulfonyl-protected 4,5,6,7-tetrahydroindol-4-one **204** was alkylated with *α*-bromoesters **205** with lithium diisopropylamide (LDA) as a base (Scheme 37) [100]. One-pot benzenesulfonyl deprotection and ester hydrolysis of **206** afforded potential antipsychotics **207** with only one diastereomer (if R = Me) isolated. Cyclization with hydrazine hydrate yielded the pyridazin-3-(2*H*)-one fused dihydroindoles **208**.

**Scheme 38:**
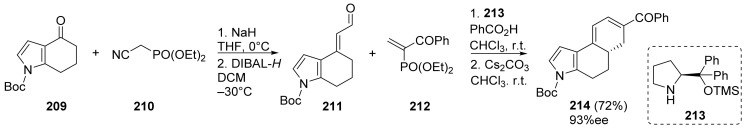
Asymmetric synthesis of six-membered carbocycles.

In 2014, Jørgensen et al. disclosed the asymmetric synthesis of optically active six-membered carbocycles, fused with a variety of ring systems [101]. This method uses an asymmetric *γ*-alkylation of enals onto olefins bearing a phosphonate substituent followed by an intramolecular Horner-Wadsworth-Emmons (HWE) reaction. Thus, enal **211** was prepared from the corresponding Boc-protected 4,5,6,7-tetrahydroindol-4-one **209** via a HWE reaction with diethyl cyanomethylphosphonate **210** and sodium hydride followed by DIBAL-*H* reduction of the nitrile to the aldehyde (yield was not reported) (Scheme 38). The asymmetric *γ*-alkylation of enal **211** with electrophile **212** was catalyzed with a trimethylsilyl (TMS) protected diphenylprolinol **213** via its dienamine intermediate with the bulky substituent of **213** shielding one side of the nucleophilic center. Next, through addition of Cs_2_CO_3_, an intramolecular HWE reaction occurred in a one-pot fashion affording the optically active six-membered carbocycle **214** in 72% yield with a remarkable enantioselectivity of 93%.

## 7. Synthesis of Spiro Compounds

**Scheme 39:**
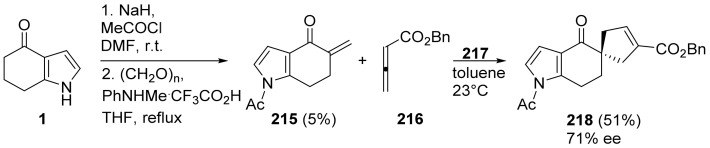
Enantioselective synthesis of spiro compound **215**.

The synthesis of spiro compounds is of great importance in view of their medicinal applications. In 2007, Miller et al. reported an enantioselective formal [3 + 2]-cycloaddition of allenoate esters with enones which was catalyzed with Boc-protected diphenylphosphine amino ester **217** (Scheme 39) [102]. Mechanistically, it is thought that the Lewis basic phosphine forms a zwitterionic dipole with the allenic ester which can undergo a [3 + 2]-cycloaddition onto enones. The effect of various *N*-substituents on the catalyst on the reaction output suggested that it is required that the N-H forms a hydrogen bond in the transition state. Furthermore, the approach of the dipolarophile is at the opposite site compared to the bulky diphenyl phosphine. Thus, enone **215** was prepared from 4,5,6,7-tetrahydroindol-4-one **1** via acetyl protection and subsequent Mannich/elimination reaction with paraformaldehyde and *N*-methylanilinium trifluoroacetate. However, the yield was only 5% over two steps. Next, reaction of **215** with benzyl allenic ester **216** with catalytic amount of **217** afforded **283** in 51% yield and an enantioselectivity of 71%.

## 8. Miscellaneous

### Rearrangements

**Scheme 40:**
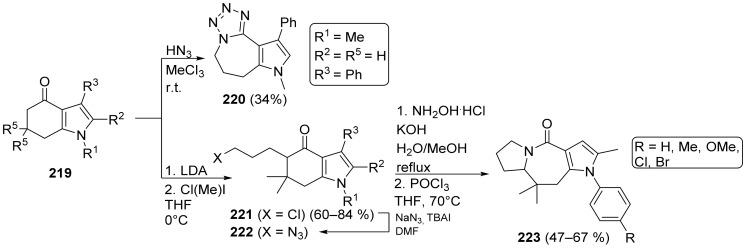
Beckmann rearrangements with the formation of polyheterocyclic structures.

Many examples can be found in literature where pyrrole-fused azepinones are formed through Beckmann or Schmidt rearrangements. In this review, we focus on Beckmann or Schmidt rearrangements with the formation of polyheterocyclic structures. In 1978, Bardakos and Sucrow reported the formation of tetrazole **220** through the Schmidt reaction of **219** with an excess of hydrazoic acid in chloroform in 34% yield (Scheme 40) [103]. Interestingly, only one regioisomer was observed. An intramolecular Schmidt reaction was attempted with **222**, however, it did not afford the envisioned product **223** (Scheme 40) [104]. The Beckmann ring expansion with hydroxylamine and POCl_3_ followed by intramolecular substitution of the chloride did afford tricyclic compound **223** in 47–67% yield. Recently, azides **222** have shown to be potential inhibitors of the SARS-CoV-2 main protease [105]. Biological activities of these tricyclic pyrrole-fused azepinones **223** are yet unknown.

**Scheme 41:**
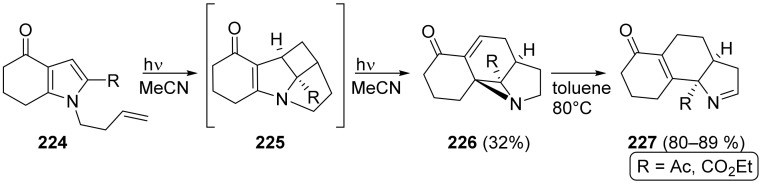
Photoinduced cycloaddition and rearrangement followed by a thermal [1,5]-hydrogen shift.

In 2013, the group of Booker-Milburn disclosed the synthesis of complex tricyclic and tetracyclic (in the case of tetrahydroindol-4-one **224**) aziridines from the photo-induced rearrangement of pyrroles [106]. Thus, *N*-butenyl substituted pyrroles were irradiated with a 6 W low pressure mercury lamp (with most of the emission centered at 254 nm and a small amount of radiation at 312 nm) inducing intramolecular [2 + 2]-photocycloaddition forming a cyclobutane-fused dihydropyrrole. In cyclobutanes bearing an acyl group at C4 of the pyrrole, such as **225**, further excitation leads to biradical bond cleavage and rearrangement to the corresponding aziridine **226** (Scheme 41). Furthermore, it was demonstrated that scale-up of these reactions could be achieved through flow chemistry. In their research concerning the synthetic possibilities of these complex aziridine rings, Knowles and Milburn reported the unusually facile thermal homodienyl-[1,5]-hydrogen shift reaction of **226** [107]. The formed tetracyclic aziridines **226** were unstable and spontaneously underwent the following thermal [1,5]-hydrogen shift reaction at room temperature toward **227**, which explains the moderate yield of **226** due to purification issues. The scope of the reaction was investigated and utilized for kinetic studies which suggest that the high reaction rate of some substrates is due to a combination of ring strain and a highly rigid conformation.

## 9. Conclusions

The inherent reactivity of the pyrrole and ketone functionality make 4,5,6,7-tetrahydroidol-4-ones valuable building blocks for the synthesis of medicinally or spectroscopically interesting structures. In this review, a brief overview of the strategies toward 4,5,6,7-tetrahydroindol-4-ones is given. Afterward, we discussed the use of this multifunctional building block for the construction of polyheterocyclic structures which were categorized based on the size and attachment point of the newly formed (heterocyclic) ring. Medicinal or spectroscopic applications were briefly mentioned.

In general, most [1,2]-fused polyheterocyclic structures are originating from an intramolecular electrophilic aromatic substitution (or formal C-H activation) with an *N*-substituent bearing an electrophilic functionality. This strategy is mostly followed due to the inherent nucleophilicity of the C-2 position of the pyrrole and used for the synthesis of five-, six- and seven-membered (hetero)cycles. The electrophilic functionalities explored are halogens, carbonyls, allenes and alkynes. Other examples are the use of a Dieckmann condensation and a three-component reaction with an unidentified mechanism, however, most likely an oxidation step with air or HNO_2_ is involved.

When both the N-H of the pyrrole and the C-2 position are substituted, the C-3 position shows nucleophilicity. Therefore, synthesis of [2,3]-fused polyheterocycles from 4,5,6,7-tetrahydroindol-4-ones are mostly starting from an N-substituted pyrrole with the C-2 bearing an electrophilic functionality, which can be introduced on the C-2 position via an electrophilic aromatic substitution or also with formal C-H activation. We discussed the intramolecular electrophilic aromatic substitution on the C-3 as well as double electrophilic aromatic substitutions and C-H activations, first on the C-2 and subsequently on the C-3 position. In the literature, we found multiple examples of six-membered rings fused onto the [2,3]-position of 4,5,6,7-tetrahydroindol-4-ones. However, research onto the synthesis of other ring-sizes fused onto the [2,3]-position is limited by one example where a five-membered fused ring was obtained in one step starting from 3-aminocyclohex-2-enones and ninhydrin.

Due to the considerable ring strain of [3,4]-fused indoles, reports involving tetrahydroindolone are rare. Only one example is found of a five-membered ring [3,4]-fused to an indole, obtained in multiple steps from tetrahydroindolone, which was an unexpected outcome of the reaction. Six- and seven-membered rings are also reported, often in multistep procedures. These [3,4]-fused six-membered rings are interesting structures resembling derivatives of ergoline alkaloids with potential medicinal applications.

The enolizable ketone functionality of 4,5,6,7-tetrahydroindol-4-ones is an excellent starting point to form [4,5]-fused indoles. This includes pyrroles, pyrazoles, 1,2,3-triazoles, isoxazoles, thiazoles, thiophenes and indoles for five-membered rings as well as (thio)pyranones, pyridinones, pyridazinones, pyrimidine and a carbocycle for the six-membered rings. Interestingly, dehydrogenation of the [4,5]-fused dihydroindole provides the fused indole. However, depending on the reaction conditions, aromatization can occur already during the attempted formation of the fused dihydroindole. Many of these fused indoles have been explored as potentially interesting biological compounds. Therefore, we are convinced that many new routes toward these [4,5]-fused indoles will be explored in the near future.

Interestingly, only one example of the synthesis of spiro compounds starting from tetrahydroindolones can be found. It can be stated that to further explore the chemical space in biologically relevant indolones, more research should be devoted to the exploration of these spiro compounds.

Lastly, a Beckmann and Schmidt rearrangement was discussed to expand the six-membered ring of tetrahydroindolones to obtain seven-membered rings fused to indoles. Another interesting report uses a light-promoted intramolecular [2 + 2]-cycloaddition of *N*-allyl tetrahydroindolone to obtain an instable cyclobutane ring which could further rearrange to interesting tetracyclic aziridines.

## Data Availability

Not applicable.
